# MHD instability dynamics and turbulence enhancement towards the plasma disruption at the HL-2A tokamak

**DOI:** 10.1038/s41598-023-31304-5

**Published:** 2023-03-23

**Authors:** Y. C. Li, M. Jiang, Y. Xu, Z. B. Shi, J. Q. Xu, Yi. Liu, A. S. Liang, Z. C. Yang, J. Wen, Y. P. Zhang, X. Q. Wang, Y. J. Zhu, H. Zhou, W. Li, Y. Luo, X. Su, HL-2A, X. R. Duan, X. T. Ding, J. Q. Dong, Q. W. Yang, L. W. Yan, Yi Liu, X. L. Zou, D. Q. Liu, W. M. Xuan, L. Y. Chen, J. Rao, X. M. Song, W. C. Mao, Q. M. Wang, Z. Cao, B. Li, J. Y. Cao, G. J. Lei, J. H. Zhang, X. D. Li, S. J. Wang, A. D. Liu, M. N. Bu, Y. H. Chen, W. Chen, J. Cheng, C. H. Cui, Z. Y. Cui, Z. C. Deng, Y. B. Dong, B. B. Feng, Q. D. Gao, X. Y. Han, W. Y. Hong, H. T. Hu, M. Huang, Y. Huang, X. Q. Ji, Z. H. Kang, T. Lan, G. S. Li, H. J. Li, Qing Li, Qiang Li, W. Li, Y. G. Li, Z. J. Li, Z. T. Liu, C. W. Luo, X. H. Mao, Y. D. Pan, J. F. Peng, K. Shao, X. Y. Song, H. J. Sun, A. K. Wang, H. Wang, M. X. Wang, Y. Q. Wang, Z. T. Wang, W. W. Xiao, Z. G. Xiao, Y. F. Xie, L. H. Yao, L. Y. Yao, D. L. Yu, B. S. Yuan, K. J. Zhao, Y. Z. Zheng, G. W. Zhong, C. P. Zhou, J. Zhou, Y. Zhou, J. C. Yan, C. X. Yu, C. H. Pan, Yong Liu

**Affiliations:** 1 Institute of Fusion Science, School of Physical Science and Technology, Southwest Jiaotong University, Chengdu, 610031 People’s Republic of China; 2 Southwestern Institute of Physics, P. O. Box 432, Chengdu, 610041 People’s Republic of China; 3 University of Science and Technology of China, Hefei, People’s Republic of China; 4 Association Euratom-CEA, IRFM, CEA, Cadarache, 13108 Saint Paul Lez Durance, France

**Keywords:** Energy science and technology, Physics

## Abstract

The evolutions of MHD instability behaviors and enhancement of both electrostatic and electromagnetic turbulence towards the plasma disruption have been clearly observed in the HL-2A plasmas. Two types of plasma disruptive discharges have been investigated for similar equilibrium parameters: one with a distinct stage of a small central temperature collapse ($\sim$ 5–10%) around 1 millisecond before the thermal quench (TQ), while the other without. For both types, the TQ phase is preceded by a rotating 2/1 tearing mode, and it is the development of the cold bubble from the inner region of the 2/1 island O-point along with its inward convection that causes the massive energy loss. In addition, the micro-scale turbulence, including magnetic fluctuations and density fluctuations, increases before the small collapse, and more significantly towards the TQ. Also, temperature fluctuations measured by electron cyclotron emission imaging enhances dramatically at the reconnection site and expand into the island when approaching the small collapse and TQ, and the expansion is more significant close to the TQ. The observed turbulence enhancement near the X-point cannot be fully interpreted by the linear stability analysis by GENE. Evidences suggest that nonlinear effects, such as the reduction of local $E_r\times B$ shear and turbulence spreading, may play an important role in governing turbulence enhancement and expansion. These results imply that the turbulence and its interaction with the island facilitate the stochasticity of the magnetic flux and formation of the cold bubble, and hence, the plasma disruption.

## Introduction

Plasma disruptions, which may induce drastic heat load, electromagnetic force and energetic runaway electrons onto the plasma facing component, is one of the major concerns for the steady operation of the future nuclear fusion reactors such as ITER^1^. Improving physical understanding of the dynamics preceding the disruption can lead to better prediction, avoidance and mitigation/suppression of the disruption in the existing fusion devices and further extrapolation to ITER.

Mode locking, either induced by overlapping of multiple rotating precursor modes or error field, is one of the main causes for the disruption, as reported in JET^2,3^, NSTX^4^ and DIII-D^5,6^. Besides, many other factors such as high density^7–9^, low safety factor^10^ and high $\beta$^11^, can also induce plasma disruption. The MHD activities prior to the disruption, excited either by mode locking, massive gas injection (MGI), or by density limit, have been investigated by MHD simulations with radiation term included^12,13^ and experiments as well^5–7,14–16^. It is found that the interaction between *m*/*n*=1/1 (*m* and *n* are the poloidal and toroidal mode numbers, respectively) and 2/1 modes causes the stochasticity of the magnetic surface in the $q\le$2 (*q* is the safety factor) region and subsequently the whole outer region^7,12,17^. Experimental results in JET^14^ and DIII-D^6^ indicate that the overlap of islands with different helicities and the alignment of multiple O-points on the outboard midplane in certain toroidal plane can also lead to the thermal quench (TQ). In addition, it has been observed that an internal *m*=1 convection structure (so-called cold bubble) always presents just before the final broadening of the current profile^9,12,13,15,16^.

It is commonly observed by simulations^18–21^ and experiments^22–31^ that the magnetic island can regulate the turbulent transport by changing the local flow shear and pressure gradient. It has been found that enhanced turbulence at the separatrix can facilitate the stochastisation of the field lines and modifiy the island width^32,33^. Although it is deemed that turbulence may increase the reconnection rate and hence precipitate the disruption^32,34^, up to now, only a few evidences in TEXTOR^7^ and KSTAR^35^, have revealed the change of turbulence prior to the disruption.

In this work, two types of plasma disruption have been observed in the HL-2A low density ohmically heated plasmas, namely, one with a small central temperature collapse before TQ, while the other without. The MHD behaviors prior to the TQ, including the evolutions of the 2/1 tearing mode and 1/1 cold bubble, have been clearly detected by the 2D advanced electron cyclotron emission imaging (ECEI) diagnostic. In addition, the micro magnetic, temperature and density fluctuations all increase towards the small collapse, and even more explosively towards TQ phase. Meanwhile, the 2D distribution of micro-$T_e$ fluctuations indicates that the turbulence near X-point increase drastically and expands into the island region just before the small collapse and the TQ. It is found that the observed turbulence enhancement cannot be fully explained by the linear stability analysis (gradient-driven mechanism), but could be related to the reduction of the flow shear and turbulence spreading effect. These results suggest that the turbulence and its interaction with the island play an important role in the stochasticity of the magnetic flux and formation of the cold bubble, and consequently, promotes the energy quench.

Following the introduction part, the rest of this paper is organized as follows: the experimental set-up is given in “Experimental setup” section. The experimental results and discussions, including (i) observations of a small heat collapse before the plasma disruption, (ii) MHD instability dynamics towards the small collapse and the TQ, (iii) turbulence enhancement towards the small collapse and the TQ, and (iv) the possible role of turbulence in facilitating the small collapse and TQ, are presented in “Experimental results and discussions” section. Finally, a summary is given in “Summary”.

**Figure 1:**
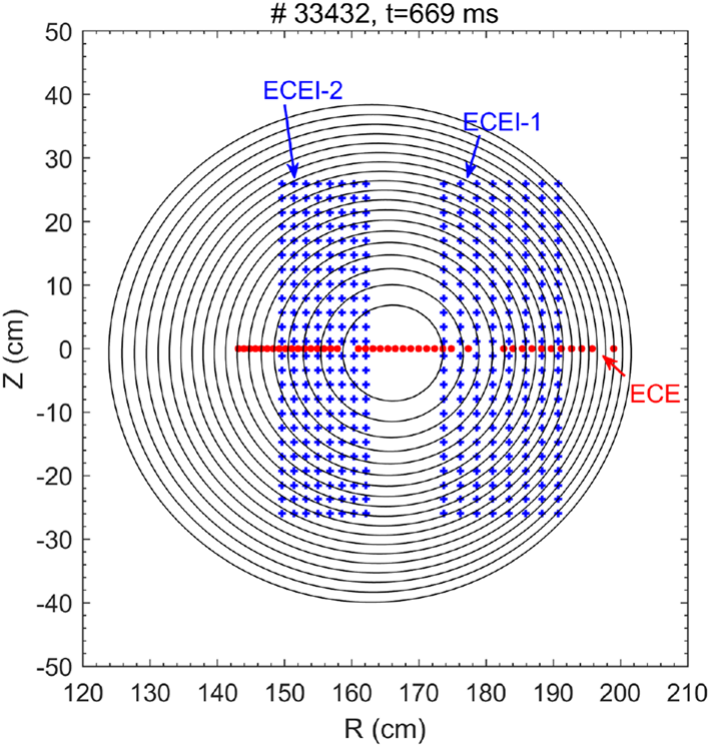
Measurement points of two ECEI arrays and the ECE diagnostic. The black circles denote the magnetic flux surfaces at $t=669$ ms in shot #33432.

## Experimental setup

The experiments were executed at the HL-2A tokamak,which is a medium-sized tokamak with a major radius $R_0$ = 1.65 m and minor radius *a* = 0.4 m. The plasma was ohmically heated under a limiter configuration.

The electron temperature ($T_e$) profile was measured by the 60-channel electron cyclotron emission (ECE) radiometer in optically thick plasmas with a temporal resolution of 2 $\mu s$ and spatial resolution of 1 cm^36^, while the absolute $T_e$ value was obtained by cross calibration with core channels of the Thomson scattering diagnostic. Two 24 (vertical) $\times$ 8 (radial) ECEI arrays were used to simultaneously measure the macro-scale $T_e$ perturbations and micro-scale $T_e$ fluctuations with a sampling rate of 500 kHz and spatial resolution of 1.8–2.3 cm^37,38^. The HL-2A ECEI system has provided comprehensive information about the 2D spatial structure of the MHD instabilities (such as tearing modes^22^, Alfv$\acute{e}$n eigenmodes^39,40^, and quasi-interchange mode^41^) and distribution of the $T_e$ turbulence^23^. The ECE and ECEI diagnostics are installed at the same toroidal position of the HL-2A device. Figure 1 illustrates the measurement locations of ECE and ECEI for shot 33432, where the black circles denote the magnetic flux surfaces obtained by the two dimensional MHD equilibrium code EFIT^42^ at 669 ms. The eight-channel far infrared (FIR) interferometer-polarimeter, horizontally located at Z $=\pm$ 3.5, ± 10.5, ± 17.5 and ± 24.5 cm, was used to detect the density profile and Faraday rotation angle^43^ in the plasma core region. The edge density profile was measured by a frequency modulated continuous wave (FMCW) reflectometer operated in extraordinary mode polarization with a spatial resolution of 0.5–1 cm^44^. The perpendicular rotation velocity ($V_\perp$) and density fluctuations with characteristic perpendicular wavenumber ($k_\perp$) were measured by the X-mode and O-mode Doppler backscattering (DBS) reflectometry^45^, with the working frequency of 31–48 GHz. It was installed 12 cm below the midplane. The time derivative of the magnetic perturbation and the poloidal (*m*) and toroidal (*n*) mode numbers of the perturbation (dominated by $m/n=2/1$) are detected by Mirnov coils, with 18 coils distributed poloidally and 10 coils distributed toroidally around the device wall.

**Table 1:** List of the shotnumbers and parameters ($I_p$, ${\bar{n}}_e$ and $q_{a}$) in disruptive discharges with and without a small collapse before the TQ.

	Shotnos	$$I_p$$ (kA)	$${\bar{n}}_e$$ (10$$^{19}$$m$$^{-3})$$	$$q_{a}$$	$$\Delta T_e/T_e$$	$$\Delta I_p/I_p$$
With small collapse before TQ	33432, 33439 33427	140–150	0.8	4.1–4.2	5–10%	10–15%
W/o small collapse before TQ	33433, 33429 33484	140–150	0.8–0.95	4.1–4.3		10–15%

The last two columns are the relative reduction of temperature ($\Delta T_e/T_e$) caused by the small collapse and plasma current ($\Delta I_p/I_p$) by partial disruption (CQ phase), respectively.

## Experimental results and discussions

### Observations of a small heat collapse before the plasma disruption

Two types of plasma disruptive discharges are observed, i. e., with and without a small central temperature collapse before the thermal quench (TQ). Both types have similar plasma equilibrium parameters: plasma current ($I_p$) is about 140–150 kA, central line-averaged density (${\bar{n}}_e$) is (0.8–1)$\times$ 10$^{19}$ m$^{-3}$, and the safety factor at the plasma edge ($q_{a}$) is 4.1–4.3, as shown in Table 1. Note that the plasma disruption concerned in this work is partial, with $\sim$ 40–50% of central electron temperature ($T_e$) drop during the TQ phase and 10–15% plasma current drop within 2–5 ms in the current quench (CQ) phase. The small collapse causes a central $T_e$ drop by 5–10%.

**Figure 2:**
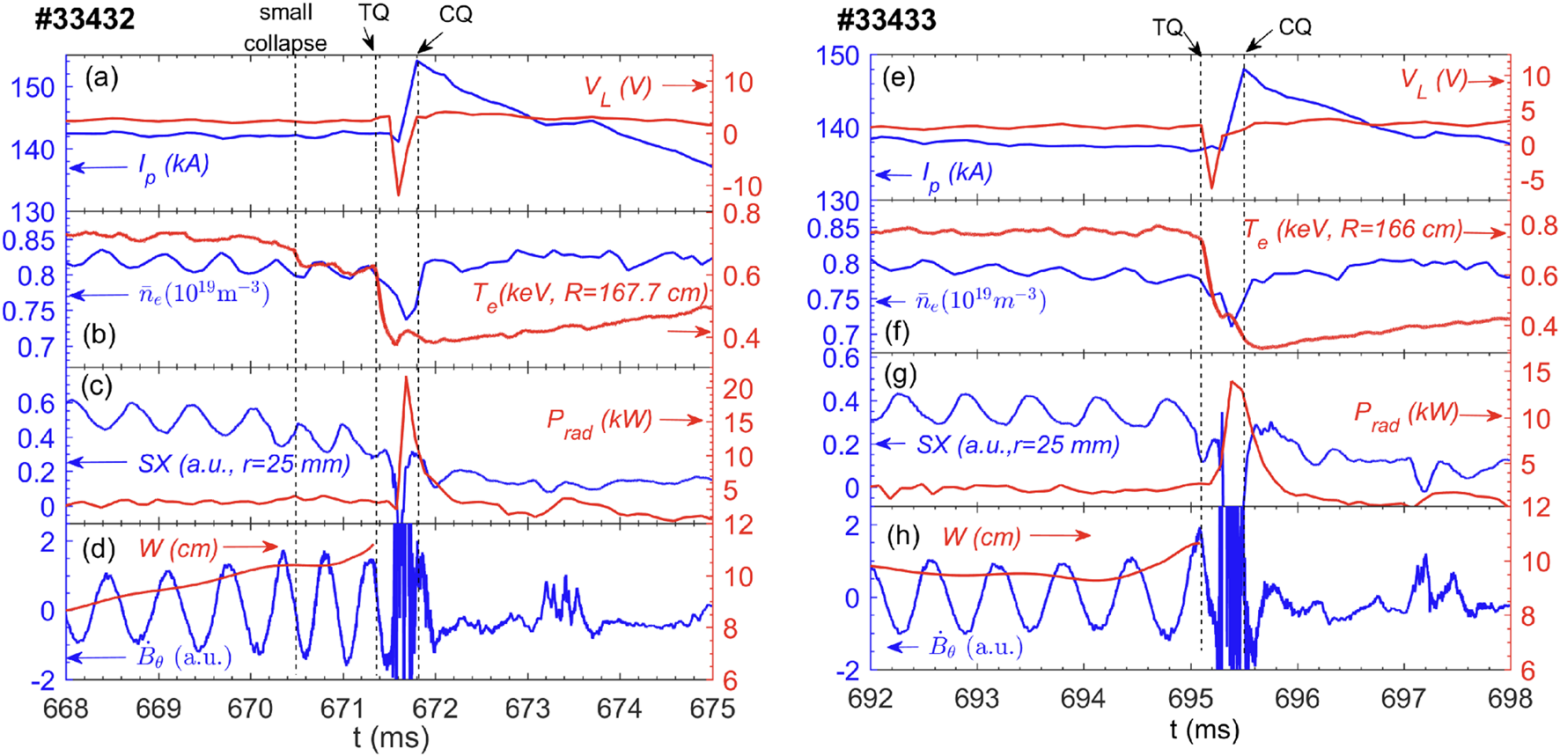
(**a**)–(**d**) Time evolutions of discharge parameters for #33432 with a small collapse prior to the TQ. The small collapse, TQ and CQ occur at 670.45 ms, 671.37 ms and 671.81 ms, respectively. (**e**)–(**h**) Time evolutions of discharge parameters for #33433 without small collapse prior to the TQ. The TQ and CQ occur at 695.1 ms and 695.5 ms, respectively. Plotted in (**a**) and (**e**) are plasma current and loop voltage, (**b**) and (**f**) central line-averaged density and core electron temperature, (**c**) and (**g**) Soft X-ray radiation with the chord through $r=25$ mm and total radiated power, (**d**) and (**h**) Mirnov coil signal ($\dot{B}_\theta$) and island width (*W*).

Figures 2a–d show the temporal evolutions of plasma parameters of shot 33432, with a small central temperature collapse occurring about 1 ms before the TQ. Here, the onsets of the small collapse, TQ and CQ take place at 670.45 ms, 671.37 ms and 671.81 ms, respectively. Note that the starts of the small collapse and TQ are both defined by the drop in the core temperature, even though a fast heat transport event induces a small $T_e$ drop in the $q\sim$2 region at about 100–200 $\upmu \, s$ prior to the core $T_e$ drop, as illustrated by the black arrows in Fig. 4 and ECEI images in Fig. 7b. The onset of the CQ phase (also the end of the TQ phase) commences with the $I_p$ spike. For comparison, Figs. 2e–h show the plasma parameters without small collapse, but only with the TQ and CQ occured at 695.1 ms and 695.5 ms, respectively. Both shots have evident oscillations in the plasma density, soft X-ray (SX) and Mirnov signals before the TQ phase, and a core $T_e$ drop by $\sim$ 40-50% within $\sim$ 200 $\mu s$ during the TQ phase. The negative loop voltage as well as the $I_p$ spike at the onset of the CQ indicate the broadening of the current density profile (Fig. 2a,e). Nevertheless, the CQ phase is not the focus of this study. The total radiation power detected by the bolometer surges after the temperature collapse and reaches a peak near the end of the TQ phase (Fig. 2c,g). Figures 2d,h show temporal evolutions of the time derivative of magnetic perturbations and the island width (*W*). Here, *W* is estimated from the magnetic measurement by mapping the probe data at the tokamak outboard wall to the radial magnetic field at the *q*=2 rational surface, i. e., ${\tilde{B}}_{r}(r_s)\sim (b/r_s)^{m+1}{\tilde{B}}_\theta (wall)|_{n}$^46,47^, where *b* and $r_s$ denote the minor radii of the Mirnov probe and the rational surface, respectively, and ${\tilde{B}}_\theta (wall)|_{n}$ is the integrated magnetic perturbation amplitude at the wall for *n*=1. For the calculation of *W*, the variation of local magetic shear and the location of the *q*=2 surface have already been taken into account. The absolute value of *W* is determined by the maximum radial distance of the flat area (between the island separatrices) in the phase-lock averaged $T_e$ contour measured by ECE, as illustrated in Fig. 5. For both shots, the island width increases continuously towards the TQ (see red curves in Fig. 2d,h). Note that the waveforms in SX and magetic perturbations are slightly deformed before the small collapse due to the impact of the second harmonic mode ($m=4$, as identified by ECEI, not shown here). Nevertheless, the amplitude of the $m/n=4/2$ mode is much smaller than that of the $m/n=2/1$ mode.

**Figure 3:**
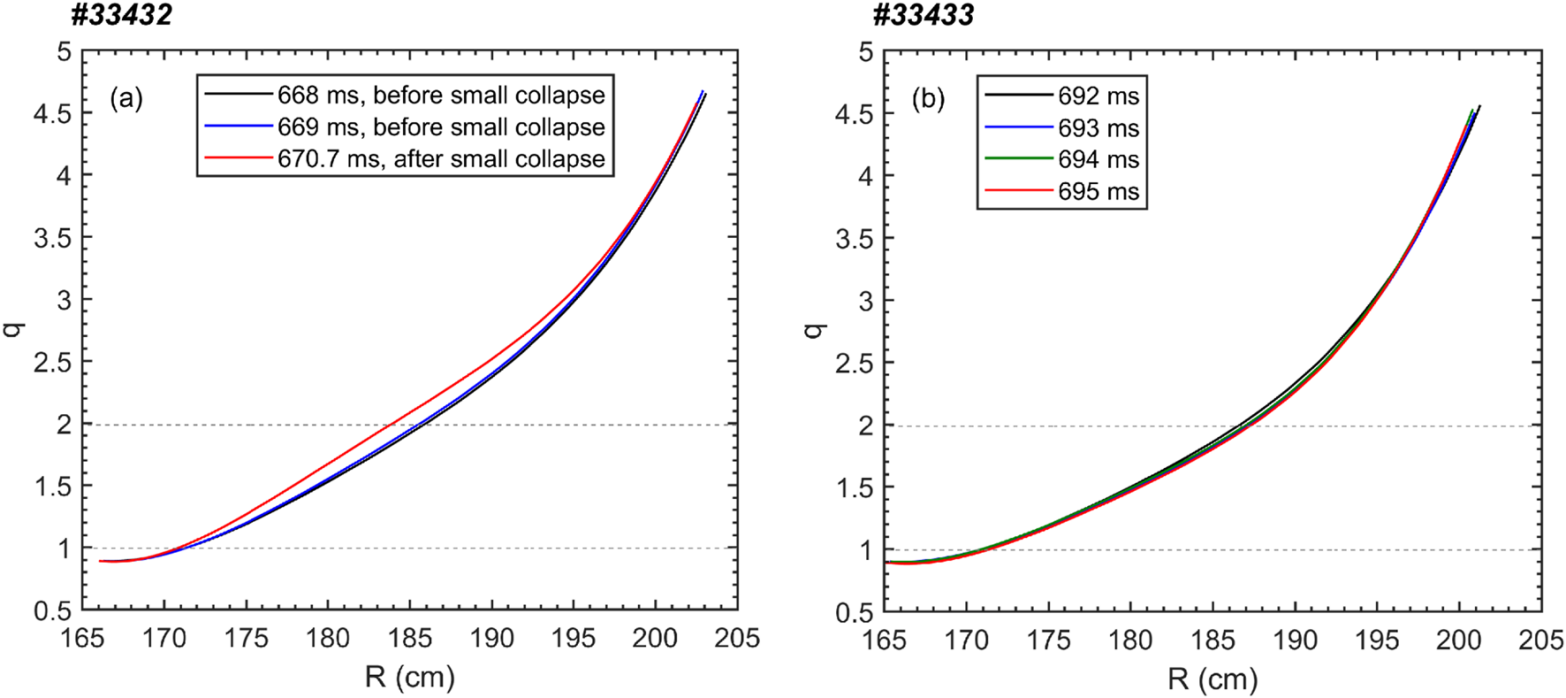
Radial profiles of *q* values at several times before the TQ for (**a**) #33432 and (**b**) #33433.

In this study, the safety factor (*q*) is estimated as follows: (i) before the TQ onset, the *q* profile is calculated by the EFIT^42^ reconstruction, which is internally constrained by the Faraday rotation angle and externally constrained by the magnetic measurement; (ii) after the TQ onset, the location of *q*=1 surface is roughly derived from the $T_e$ profile and its perturbations, which will be described in more detail later. Depicted in Fig. 3 are the *q* profiles in the low field side at serveral times before the TQ onset for (a) #33432 and (b) #33433. It can be seen that prior to the TQ the *q* profile changes slightly before/after the small collapse toward the TQ, e. g., in #33432 the location of *q*=2 surface moves inwards about 2 *cm* after the small collapse, while in #33433 the *q*=2 surface varies about 1 *cm* when approaching the TQ.

**Figure 4:**
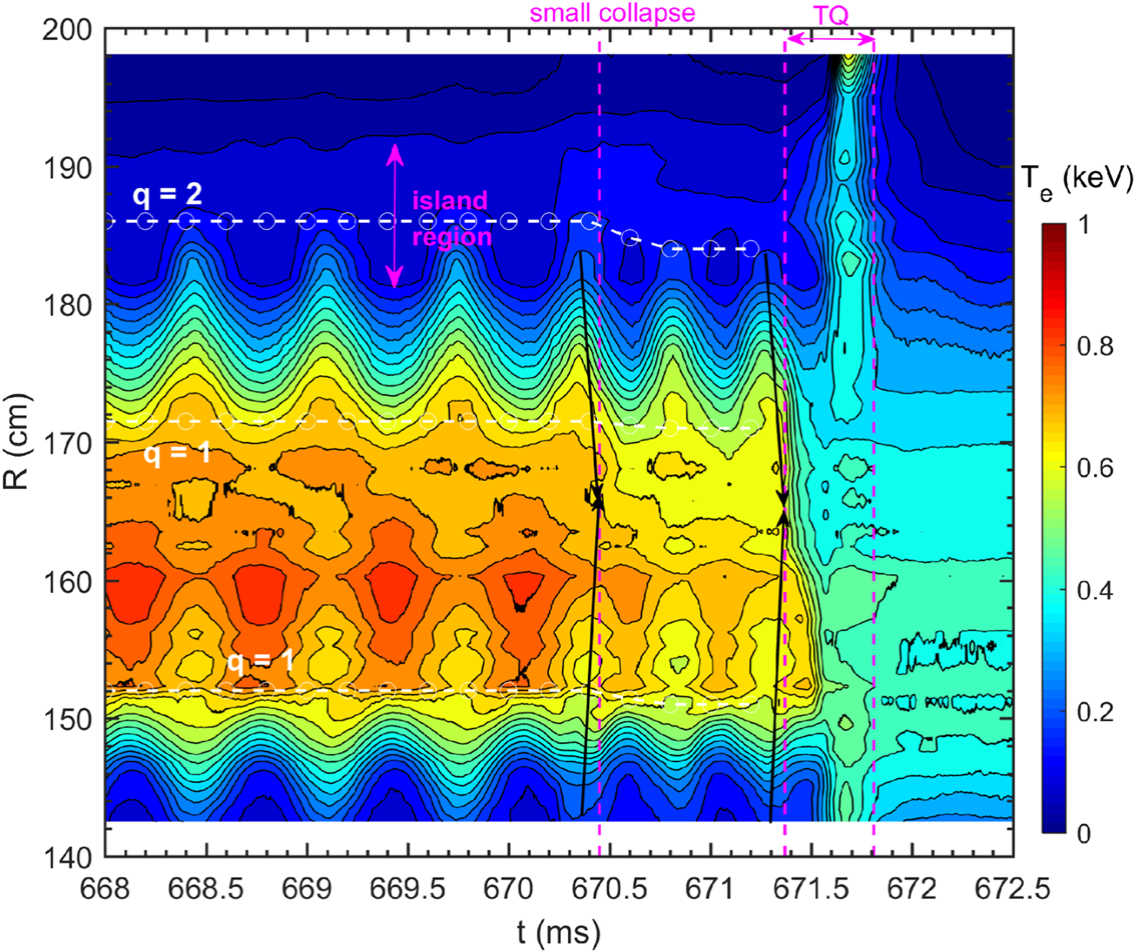
Contour plot of $T_e$ evolutions with three purple dashed lines denoting the onset of the small collapse, TQ and CQ, respectively. The 2/1 island region near 669.5 ms is marked by the double arrow. The black arrows denote the time delay between the $T_e$ drop at $q=2$ surface and in the center before the small collapse and the thermal quench (#33432).

### MHD instability dynamics towards the small collapse and the TQ

**Figure 5:**
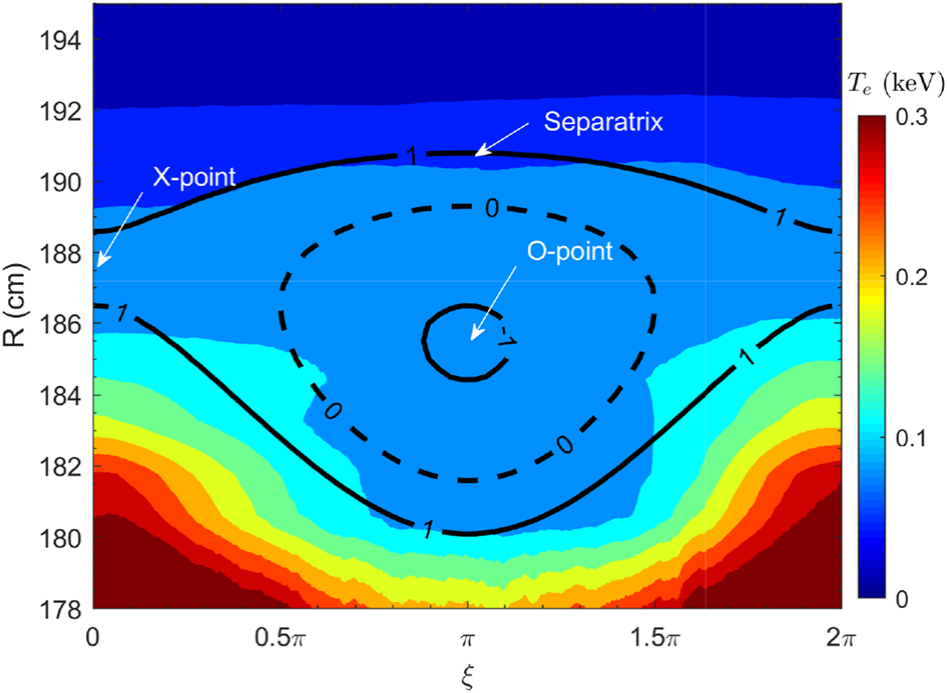
Phase-lock averaged $T_e(\xi , R)$ contour during 668.440–669.742 ms. The normalized flux surface labels at $\Omega =-1$ (O-point), 0 and 1 (separatrix) are depicted with black curves (#33432).

MHD structure and dynamics prior to the small collapse and disruption can be clearly captured by the 1D ECE radiometer and 2D ECE images by measuring the temperature profiles and perturbations with high temporal and spatial resolutions. Figure 4 shows the time evolution of the $T_e$ at different radial locations, with three purple dashed lines denoting the onset of the small collapse, TQ and CQ, respectively. Strong $T_e$ oscillations are observed near *q*=2 surface which located at $R\approx$ 186 cm in the low field side (LFS) and $R\approx$ 140 cm in the high field side (HFS), in agreement with the rotation of the 2/1 TM as identified by the Mirnov signals. The 2/1 island region is located in the range of $R\approx$ 181-191 cm in the LFS as characterized by the flat $T_e$ region marked by the purple double arrow. The detailed island structure is illustrated in Fig. 5 by the phase-lock averaged $T_e(\xi ,R)$ contour between 668.440-669.742 ms (two rotation cycles), where $\xi$ represents the helical coordinate perpendicular to the field lines. In the lab frame, $\xi =\omega _0t$, for which $\omega _0/(2\pi )\approx 1.5-2$ kHz is the island rotation frequency and *t* is the time. The black curves in the figure are the normalized flux surface labels at $\Omega =-1$ (O-point), 0 and 1 (separatrix), which denotes the flux surfaces of the magnetic island. The $\Omega (\xi ,R)$ is defined as $\Omega =8X^2+(2AX+1)\cos \xi$, where $X=(R-R_s)/W$^48^, $W$ $\approx$10 cm is the full island width, $R_s$=186 cm is the major radius of the rational surface and $A=-$0.8 is the radial asymmetry parameter^49^. Such a mapping provides more direct and accurate determination of the island helical structure and width, compared with the radial $T_e$ profile, which is limited by finite spatial resolution of the ECE radiometer.

**Figure 6:**
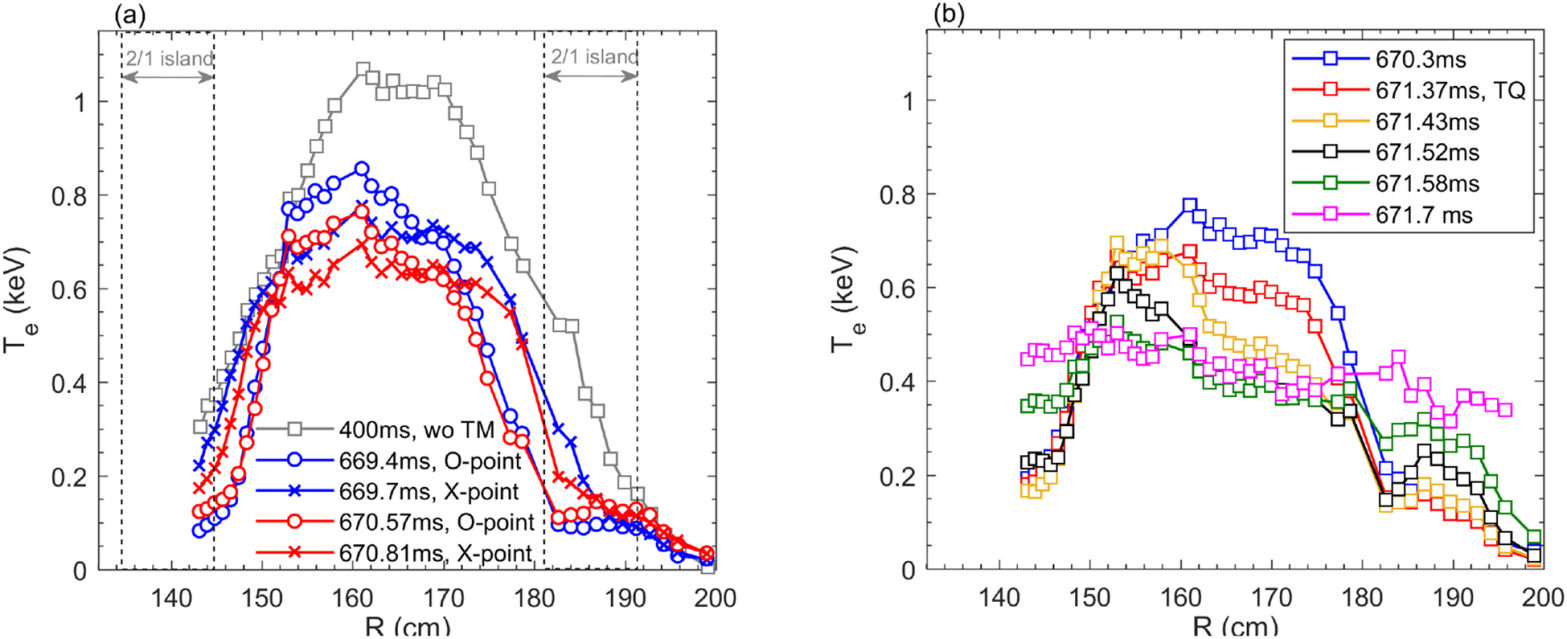
(**a**) $T_e$ profiles without tearing mode (squares), X- (blue crosses) and O-point (blue circles) phases before the small collapse, and X- (red crosses) and O-point (red circles) phases after the small collapse. The 2/1 island region is marked in the figure; (**b**) $T_e$ profiles between TQ and CQ (#33432).

Besides, the contour plot in Fig. 4 also indicates the existence of an $m=1$ structure within $R=152-{171.5}$ cm ($q=1$ surface) by the displacement of the peak in the $T_e$ profile from the original magnetic axis (also see profiles in Fig. 6a). This core helical structure, presumably 1/1 mode, rotates periodically at the same frequency as the 2/1 TM. The existence of the 1/1 mode can also be seen in the perturbation intensity of the SXR signals as well as in the amplitude and phase of the $T_e$ perturbation analyzed from the ECE signals (not shown here). The $T_e$ at the $q\sim$ 2 region drops ahead of the core $T_e$ collapse by about 100 $\upmu s$ both at the small collapse and the TQ onset times, as marked by the black arrows in the figure. Such a time delay is much shorter than the diffusive time. After the small collapse, the core $T_e$ reduces from 0.82 to 0.75 keV at $R\approx$ 160 cm. However, inside the 2/1 island, the $T_e$ increases slightly after the small collapse, which can be seen more clearly in Fig. 6a. During the TQ phase, the 1/1 mode dominates over a wider core region ($R=152-179$ cm) with the cold O-point (bubble) located in the outboard mid-plane.

Figure 6a shows $T_e$ profiles at several times, i. e., without TM at $t=400$ ms (grey squares) and across the island O-/X-point before (blue symbols) and after (red symbols) the small collapse. The core $T_e$ reduces significantly during the time period with TM compared to that without TM. The *m*=1 plasma motion leads to the inward displacement of the $T_e$ peak during the 2/1 island O-point phases (see blue and red circles), indicating an interplay between the 2/1 TM and the 1/1 internal mode. In addition, after the small collapse, the core $T_e$ decreases by 5–10%, by comparing red circles (crosses) and blue circles (crosses) in the region of $R=154-172$ cm. But the $T_e$ inside the 2/1 island increases slightly as seen from the blue and red circles in the region of $R=182-191$ cm and $R<145$ cm.

Evolutions of $T_e$ profiles during the TQ phase have been drawn in Fig. 6b. It exhibits the phenomenon of the asymmetric profile erosion, where the core $T_e$ collapses from the LFS but keeps unchanged in the HFS, as seen from the blue, red, yellow and black curves. The hollowness of the $T_e$ profiles is due to the *m*/*n* = 1/1 cold bubble moving into the plasma core. Similar $T_e$ profiles have been found in the earlier density limit disruptive discharges in TEXTOR^7^, JET^8^ and TFTR^9^ tokamaks. In the later phase of the TQ, e. g., after 671.58 ms, the edge $T_e$ increases significantly (green and purple curves), resulting in a much flatter $T_e$ profile, which is followed by the CQ.

**Figure 7:**
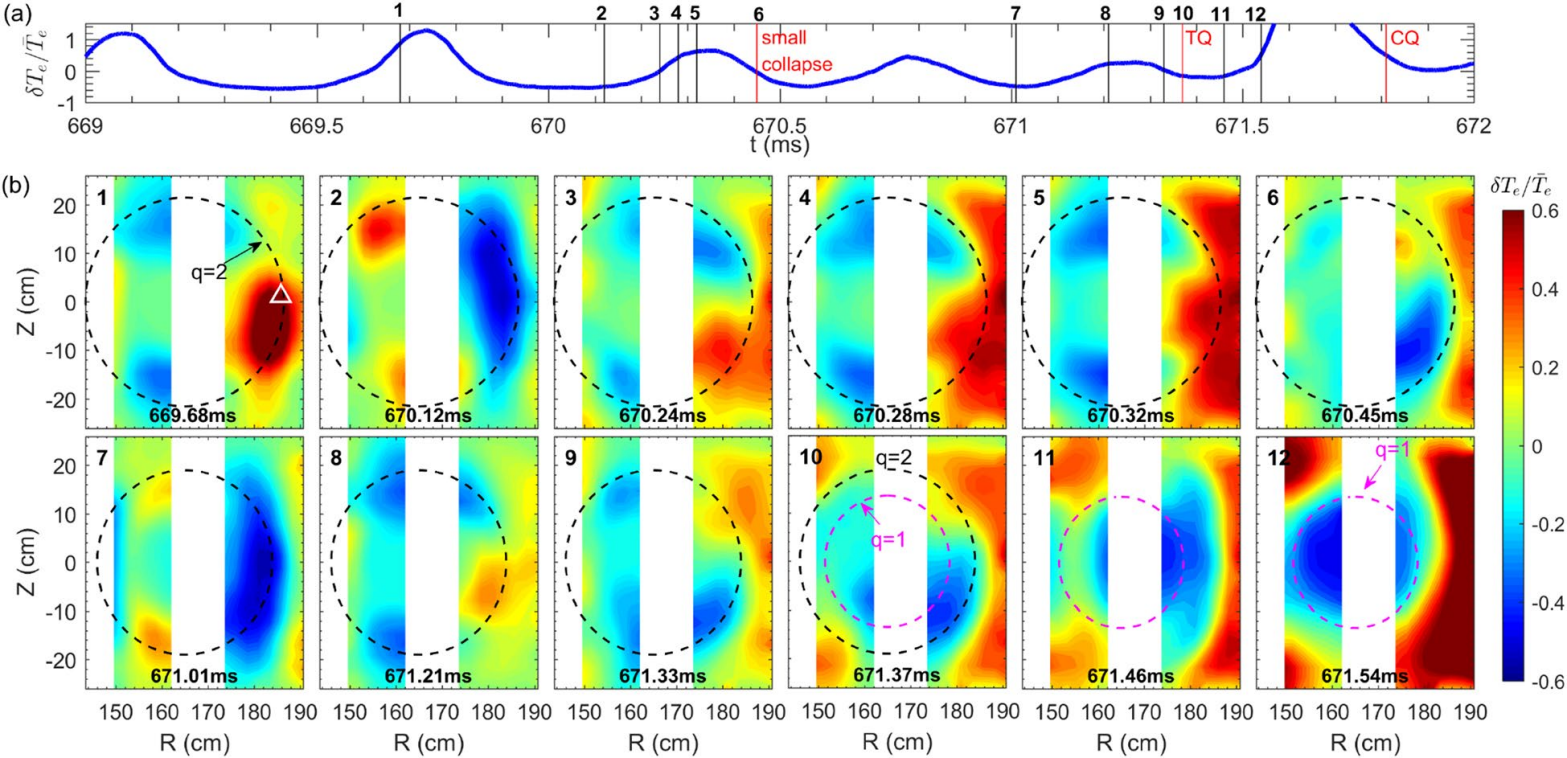
Time evolutions of the MHD instability behaviors towards the plasma disruption: (**a**) relative temperature perturbation ($\delta T_e/{\bar{T}}_e)$ measured at $R= 185.9$ cm and $Z= 1.13$ cm (marked by the white triangle in the first image of Fig. 7b). The onsets of small collapse, TQ and CQ are denoted by vertical red lines. (**b**) ECE images taken at twelves time points marked by vertical lines in Fig. 7a. The $q=2$ and $q=1$ surfaces are illustrated by black and purple dashed circles, respectively (#33432).

**Figure 8:**
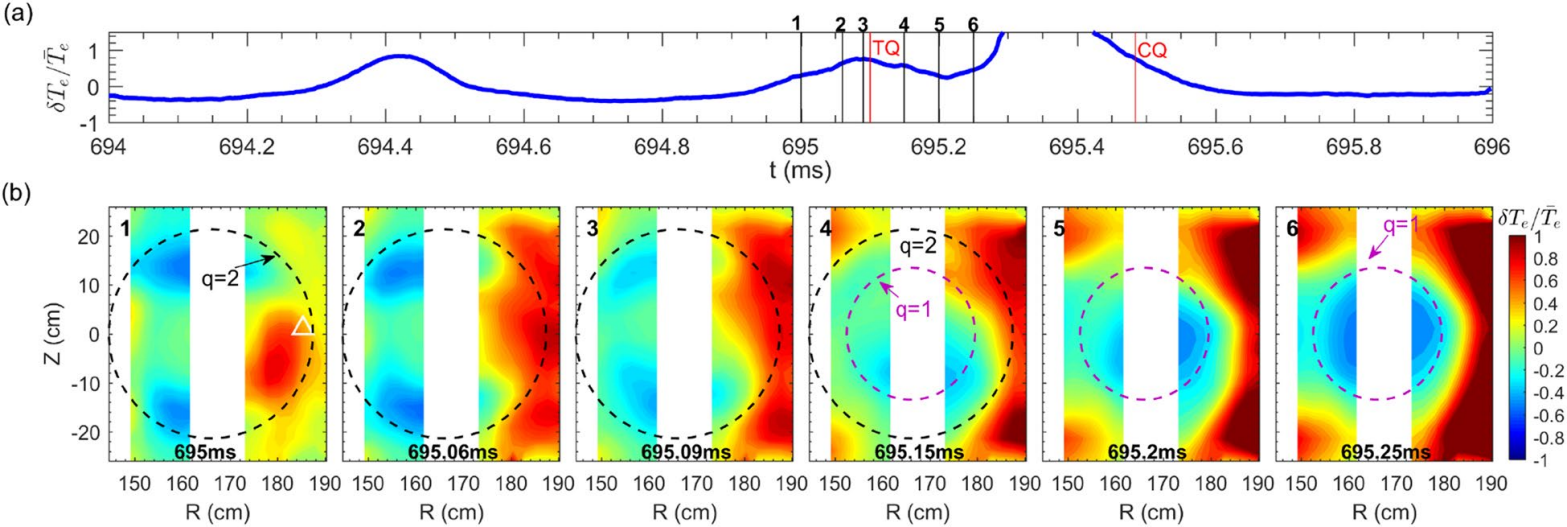
Time evolutions of the MHD instability behaviors towards the plasma disruption: (**a**) relative temperature perturbation ($\delta T_e/{\bar{T}}_e)$ measured at $R= 185.3$ cm and $Z= 1.13$ cm (marked by the white triangle in the first image of Fig. 8b). The onsets of the TQ and CQ are denoted by vertical red lines. (**b**) ECE images taken at six time points marked by vertical lines in Fig. 8a. The $q=2$ and $q=1$ surfaces are illustrated by black and purple dashed circles, respectively (#33433).

The evolutions of the 2D mode structure during the small collapse and the TQ are shown in Fig. 7. Figure 7a displays relative temperature perturbation $\delta {T}_e/{\bar{T}}_e$ ($\delta T_e=T_e-{\bar{T}}_e$, ${\bar{T}}_e$ is the average over several island rotation cycles) of one ECEI signal measured at $R=185.9$ cm and $Z=1.13$ cm (marked by the white triangle in the first image). In Fig. 7b, the $\delta {T}_e/{\bar{T}}_e$ images at 12 time points marked in Fig. 7a are illustrated. The sixth and tenth images correspond to the onset of the small collapse and the TQ, respectively. The black and purple dashed circles denote the *q*=2 and $q=1$ surfaces, respectively. Note that after the TQ onset at 671.37 ms, the response of the diagnostic (such as the polarimeter) cannot follow up the rapid evolution of the plasma parameters so that the EFIT calculation is invalid during the TQ phase. In Fig. 6b, the time evolution of $T_e$ profiles indicate that within the radial range of 152 cm $<R<$ 179 cm the $T_e$ drops gradually, whereas at the locations of $R\approx$ 152 cm and $R\approx$ 179 cm, the $T_e$ keeps almost unchanged (671.37 $\rightarrow$ 671.52 ms). Therefore, for images 10$\rightarrow$12 in Fig. 7, the *q* = 1 surface is roughly derived from these two radial locations (152 cm and 179 cm) where the $\delta T_e\approx$ 0. For #33433, the location of *q* = 1 surface for images $4\rightarrow 6$ in Fig. 8b is estimated in a similar way. Far before the small collapse, a dominant 2/1 tearing mode rotates in the electron diamagnetic drift (anti-clockwise) direction (see $2\rightarrow 4$ images). Note that, inside the *q* = 2 surface region, the hot (cold) spot denotes the inner part of the X-(O-) point of the 2/1 island^50,51^.

The heat flux begins to flow outwards through the island X-point about 200 $\upmu s$ before the small central $T_e$ collapse, as seen in images 3–6 of Fig. 7b. When close to TQ, the heat flux flows outwards again from the X-point within 150 $\upmu s$, as seen in images 8–10, similar outflow of heat flux via the X-point has been observed in sawtooth crash process in TEXTOR^52^. Meanwhile, a cold bubble develops from the inner region in one of the 2/1 island O-points at about 671.33 ms (40 $\upmu$s before the TQ), and moves toward the core region (inside $q=1$ surface) with a speed of $\sim$ 1 km/s during the TQ phase, as seen from images 9–12. The occurrence of the cold bubble is probably the cause for the TQ, but not for the small central collapse. The cold bubble developed by the interchange-like perturbation near the X-point has been detected by ECEI in the mode lock-induced major disruption in KSTAR^15^. Similar cold bubble has also been found in the MGI-induced disruptive discharges, when the impurity gas cold front penetrated into the core region^13,16,53,54^. In this study, we have found that the MHD dynamics without a small collapse before TQ are similar to those with a small collapse before the TQ. The results are illustrated in Fig. 8, where one can also see the formation (10 $\upmu$s prior to the TQ, the third image) and inward convection of the $m=1$ cold bubble. Such a cold bubble together with its motion towards the plasma core, which subsequently result in the explosive plasma energy loss, are analogous to the current-carrying ‘knot’ (occured in the condition of strong conductivity perturbation) travelling in the inward direction, as found in the earlier analytical theory^55^.

**Figure 9:**
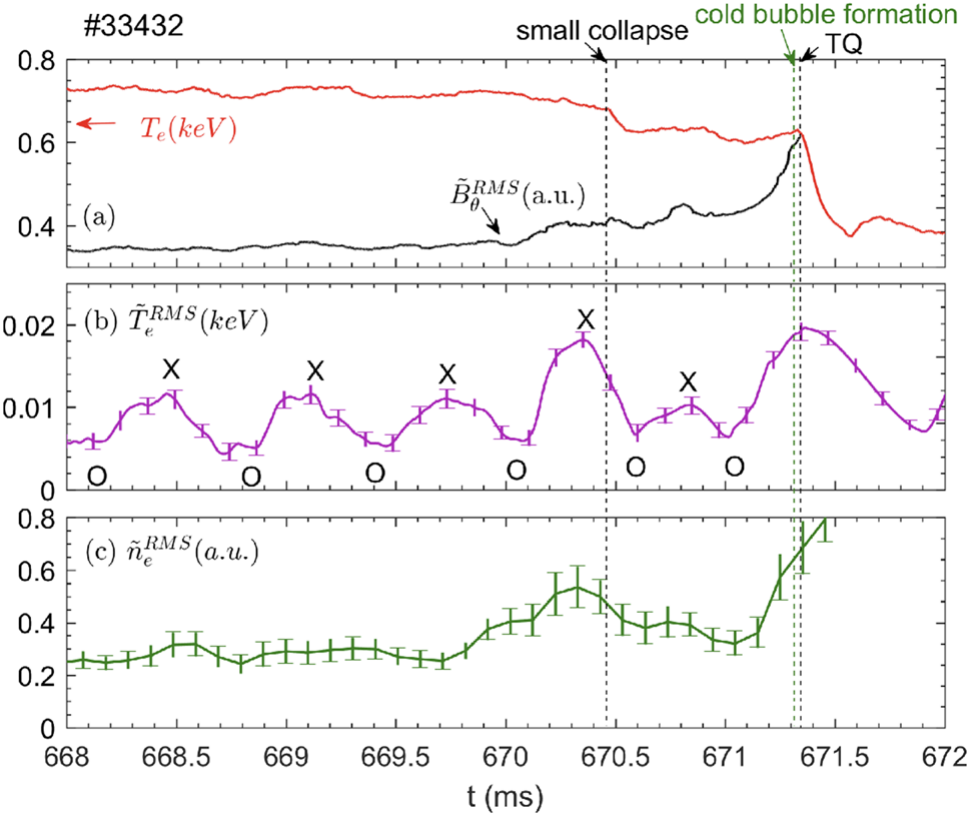
Time evolutions of (**a**) core temperature $T_e$ and magnetic fluctuation ${\tilde{B}}_\theta ^{RMS}$ in the frequency range of 15–400 kHz, (**b**) temperature fluctuation ${\tilde{T}}_e^{RMS}$ in the frequency range of 15–100 kHz and (**c**) density fluctuations ${\tilde{n}}_e^{RMS}$ in the frequency range of 15–400 kHz. The fluctuation data in (**b**) and (**c**) are estimated by averaging several channels around the island X-point. The error bars in (**b**) and (**c**) indicate the standard deviation about the mean obtained in different channels (#33432).

**Figure 10:**
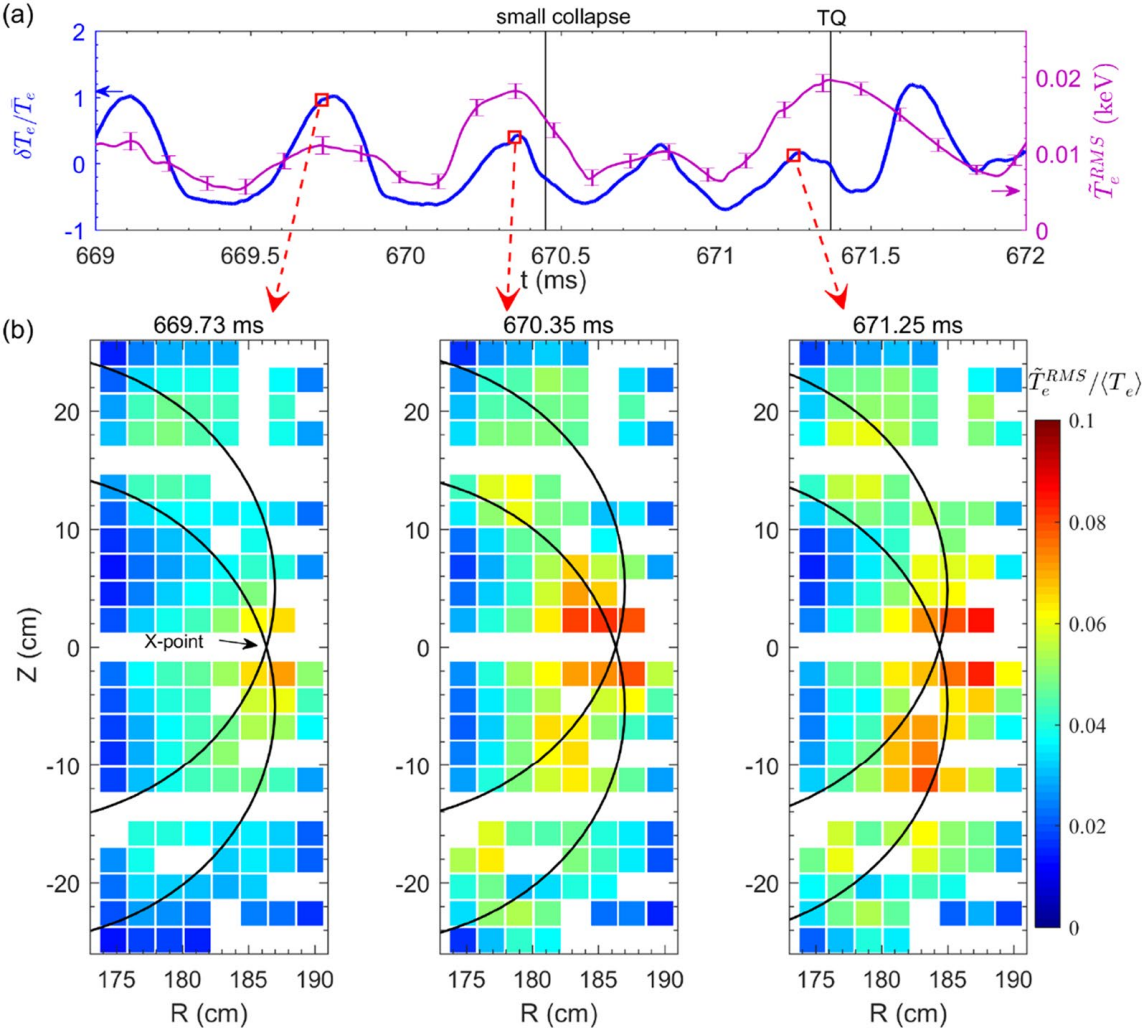
(**a**) Relative temperature perturbation $\delta T_e/{\bar{T}}_e$ (blue curve) at $R=~183.1$ cm and $Z= 3.4$ cm and ${\tilde{T}}_e^{RMS}$ obtained by averaging serveral channels of $T_e$ fluctuations (15–100 kHz) measured around the island X-point. (**b**) Two dimensional distribution of the relative RMS temperature fluctuation (${\tilde{T}}^{RMS}_e/\langle {T}_e\rangle$) in the frequency range of 15–100 kHz, at 669.73 ms, 670.35 ms and 671.25 ms, as marked by red squares in Fig. 10a. The black curves denote the island separatrices obtained by the same method as in Fig. 5 (#33432).

### Turbulence enhancement towards the small collapse and the TQ

While the interaction between the island and turbulence has been intensely investigated by simulations^18–21^ and experiments^22–31^, its role in the plasma disruption was rarely reported, except for some evidences from TEXTOR^7^ and KSTAR^35^. In our work, it is found that both the electrostatic and electromagnetic turbulence varies dramatically towards the small collapse and TQ. Figure 9 shows time evolutions of core $T_e$ and fluctuation signals for shot #33432, with (a) core $T_e$ (red curve) and edge magnetic fluctuation ${\tilde{B}}_\theta ^{RMS}$ (black curve), (b) temperature fluctuation ${\tilde{T}}_e^{RMS}$ around the *q*=2 surface, and (c) density fluctuations ${\tilde{n}}_e^{RMS}$ around the *q*=2 surface. The ${\tilde{B}}_\theta ^{RMS}$ is the root-mean-square (RMS) value of the $B_\theta$ fluctuations in the frequency range of 15-400 kHz from the Mirnov coils mounted on the outbard midplane. ${\tilde{T}}_e^{RMS}$ (${\tilde{n}}_e^{RMS}$) is obtained by averaging serveral channels of $T_e$ ($n_e$) fluctuations in the frequency range of 15-100 kHz (15-400 kHz) measured at the island X-point by ECEI (DBS). It is seen that the local $T_e$ fluctuations are modulated by the island rotation, i. e., ${\tilde{T}}_e^{RMS}$ inside the island is minimum (maximum) at the island O-point (X-point), as marked by the “O”and “X” in Fig. 9b. These results are consistent with previous observations^22–31^. Figure 9 shows that the electrostatic and electromagnetic fluctuation intensities all increase towards the small collapse, and more significantly when approaching the TQ.

Figure 10a shows time evolutions of the macro-scale $T_e$ perturbation $\delta T_e/{\bar{T}}_e$ (blue curve, $\bar{T_e}$ is the average over several island rotation cycles) to characterize the TM rotation, and micro-scale ${\tilde{T}}^{RMS}_e$ (purple curve) around the island X-point. As it is difficult to calibrate all the 2D ECEI channels in the ECE images, we use ${\tilde{T}}^{RMS}_e/\langle {T}_e\rangle$ to characterize the relative amplitude of the $T_e$ fluctuations ($k_\theta \rho _s<$ 0.7, $\rho _s$ is the ion sound Larmor radius and $k_\theta$ is the poloidal wavenumber), where the ${\tilde{T}}^{RMS}_e$ is the RMS value of $T_e$ fluctuations in the frequency range of 15–100 kHz and $\langle {T}_e\rangle$ is the ensemble average of the equilibrium $T_e$ over 100 $\upmu s$. In Fig. 10a, three time points are marked by the red squares when the island X-point passes by at 669.73 ms far before the small collapse, 670.35 ms close to the small collapse, and 671.25 ms close to the TQ. Figure 10b illustrates the 2D spatial distribution of $T_e$ fluctuations at the above three times. The black curves denote the island separatrices obtained by the same method as in Fig. 5. The white cells in the figure depict the unavailable data of the local $T_e$ fluctuation. Far before the small collapse (see the first image), strong turbulence is just localized at the X-point, while it is very small near the island O-point, consistent with the usual picture of gradient driven turbulence^22–29^. Close to the small collapse, the turbulence enhances significantly near the X-point region (also see purple curve in Fig. 10a) and expands both inwards and poloidally, and thus, mild turbulence is observed inside the island (see the second image). When approaching the TQ (see the third image), the $T_e$ turbulence further expands nearby the reconnection site (181 cm<R<189 cm) and strong turbulence is spread onto the island O-point, where the local $T_e$ gradient is close to zero. It appears that the turbulence spreads into only lower 2/1 island O-point before the TQ. A possible reason is that turbulence spreading doesn’t need gradient drive inside the island O-point, and hence, follows the diamagnetic drift direction of the fluctuation phase velocity. In our experiment, the $T_e$ fluctuations measured by the ECEI are sensitive in $k_\theta \rho _s<$0.7 range. According to the simulation results in Fig. 12b, the observed $T_e$ fluctuations are dominated by the ion temperature gradient (ITG) mode along the ion diamagnetic drift direction ($\omega _{*,i}$), which is poloidally downwards in the low field side.

**Figure 11:**
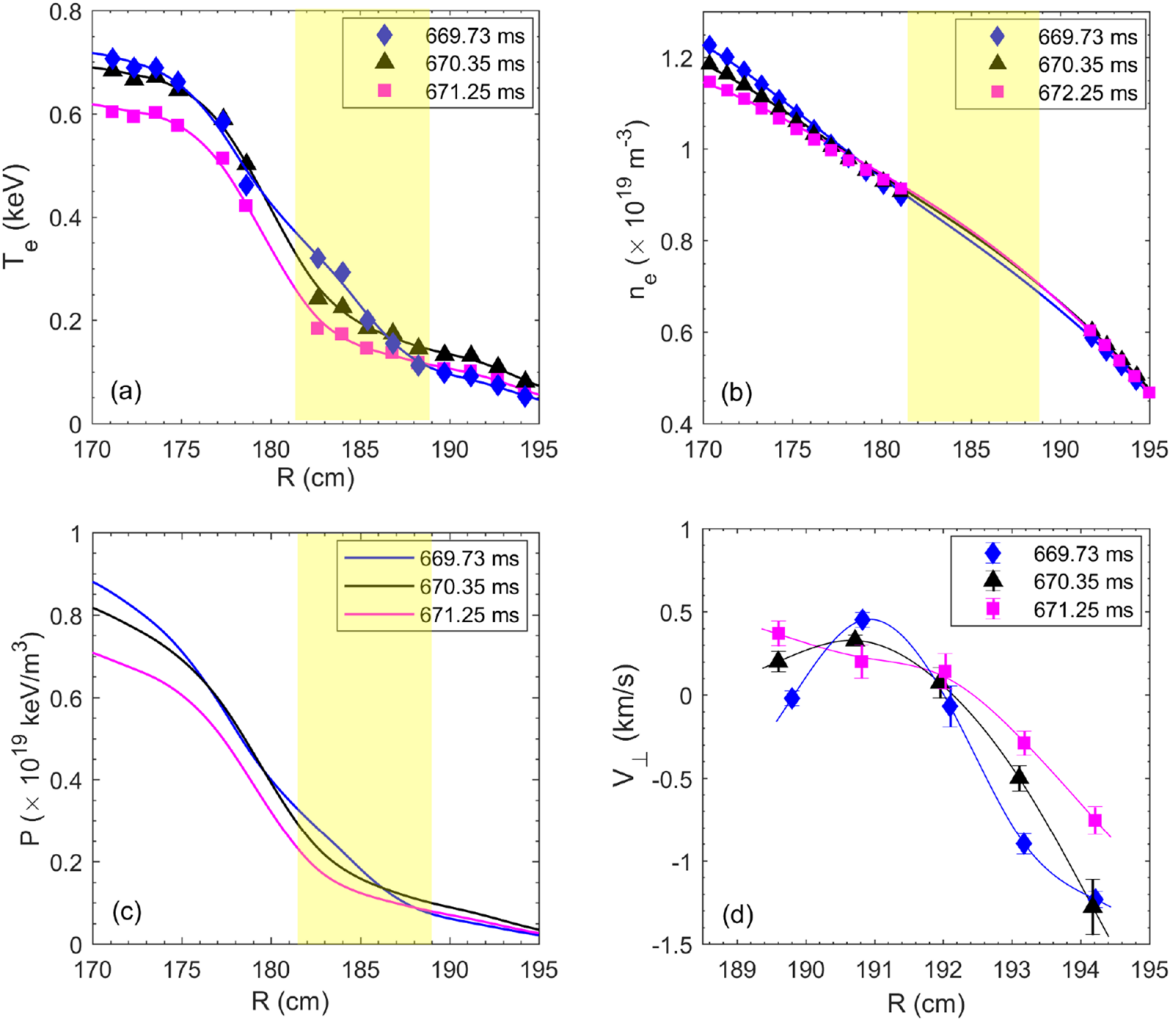
Radial profiles of (**a**) $T_e$, (**b**) $n_e$, (**c**) electron pressure *P* and (**d**) the perpendicular rotation velocity ($V_\perp$) across the island X-point at 669.73 ms, 670.35 ms and 671.25 ms, respectively. The symbols are measured data points, and the curves are from the fitting of measured data points (#33432).

**Figure 12:**
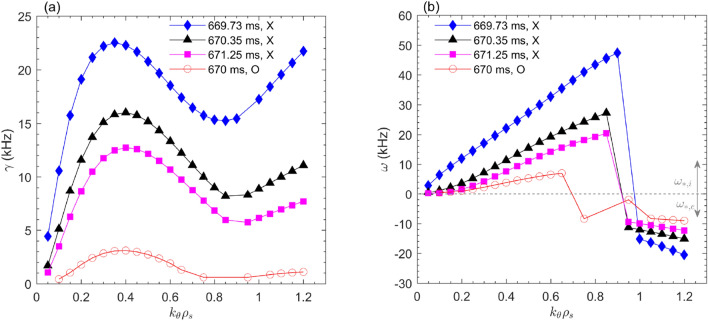
(**a**) Linear growth rates and (**b**) frequencies of the fastest growing micro-instability at $R\approx$ 185 cm during X-point (669.73 ms, 670.35 ms and 671.25 ms) and O-point (670 ms) phases. $\omega _{*,i}$ and $\omega _{*,e}$ in (**b**) denote the ion and electron diamagnetic drift direction, respectively (#33432).

Here, it is of importance to understand the mechanisms responsible for the changes of turbulence, including the driving and damping dynamics, etc. Firstly, the equilibrium parameters are compared for the above three times, i. e., 669.73 ms, 670.35 ms and 671.25 ms. Shown in Fig. 11a,b are the $T_e$ profiles measured by the ECE radiometer and density profiles measured by the FIR interferometer in the core region and FMCW reflectometer in the plasma edge. Figure 11a shows that nearby the reconnection region (181 cm <R < 189 cm marked in yellow shadow), the $T_e$ gradient is greater far before the small collapse ($t=669.73$ ms) and becomes smaller close to the small collapse ($t=670.35$ ms) and the TQ ($t=671.25$ ms). Figure 11b shows a similar tendency of density gradient in the reconnection region. These results lead to a drop of the pressure ($P(r)=n_e(r)\cdot T_e(r)$) gradient in the reconnection area from $t=669.73$ ms to $t=670.35$ ms and 671.25 ms. On the other hand, Fig. 10 reveals that the turbulence intensities near the X-point at $t=670.35$ ms and 671.25 ms are much higher than that at $t=669.73$ ms. Therefore, the observed enhancement of ${T}_e$ turbulence cannot be explained by the pressure gradient-driven mechanism.

In this study, a linear gyrokinetic simulation has been conducted by the GENE code^56^. These flux-tube simulations focus on ion scales ($k_\theta \rho _s$ = $0.1-1.2$) and use two fully kinetic species (deuterons and electrons) in collisionless plasmas with electromagnetic effects included. The input profiles are $n_e$, $T_e$, $T_i$ (not available, $T_i\approx T_e$ is assumed). The simulation predicts the growth rate of the fastest growing microinstability of the background plasma. Figure 12 depicts the growth rates and frequencies at $q=2$ region ($R\approx$ 185 cm) during X-point and O-point phases versus the wavenumber ($k_\theta \rho _s$). The $T_e$ fluctuations measured by ECEI are sensitive in $k_\theta \rho _s<0.7$ range. Therefore, the observed ${T}_e$ turbulence is dominated by the ITG mode centered at $k_\theta \rho _s\approx 0.4$ in the ion diamagnetic drift direction. The instability in the larger wavenumber range ($k_\theta \rho _s>0.9$) belongs to the trapped electron mode (TEM) propagating in the electron diamagnetic drift direction, which can be driven by either $n_e$ or $T_e$ gradient. Nevertheless, this mode has no relation with the observed turbulence in our experiments. Besides, the simulation results in Fig. 12a indicate that the growth rates at three X-points are much larger than that at the O-point, in agreement with the gradient-driven mechanism. However, among the three X-points, the maximum growth rate at 669.73 ms is contrary to the experimental observation of the least $T_e$ fluctuation magnitude, as displayed in Fig. 10.

The above results suggest that when approaching the small collapse and the TQ, the enhanced turbulence nearby the reconnection site (or X-point) is not only governed by the linear gradient-driven mechanism, but probably also by nonlinear effects^57–59^. This can be partially verified by the profiles of the perpendicular velocity $V_\perp$ measured by the DBS system at the above three X-point passing-by times, as shown in Fig. 11d. The time lag between the DBS and ECE/ECEI has been taken into account. It is reasonably assumed that the measured $V_\perp \approx V_{E\times B}$ when $V_{E\times B}\gg V_{phase}$ ($V_{phase}$ is the phase velocity of turbulence). It can be seen that close to the small collapse (*t*=670.35 ms) and the TQ (*t*=671.25 ms), the flow shear near the X-point ($R<192$ cm) is much smaller than that at *t*=669.73 ms. In the region of 181 cm<R<189 cm, although direct information of the flow shear cannot be attained from the $V_\perp$ profile, one can roughly infer the $E_r\times B$ sheared flow from the pressure gradient ($\nabla _r P$) assuming $E_r\propto \nabla _r P$ by the radial force balance, which is in good agreement with experimental measurements in tokamaks and stellarators^60,61^. It is seen from Fig. 11c that in the radial range of 181 cm<R<189 cm there are clear drops of $\nabla _r P$ from *t*=669.73 ms (blue curves) to *t*=670.35 ms (black curves) and *t*=671.25 ms (purple curves), suggesting reductions of the $E_r\times B$ flow shear when approaching the small collapse and the TQ. Note that such a drop in the $E_r\times B$ flow shear plays a significant role in the increase of turbulence and turbulence spreading, as predicted by theoretical models^62,63^ and by the experimental observation as well in LHD^64^.

**Figure 13:**
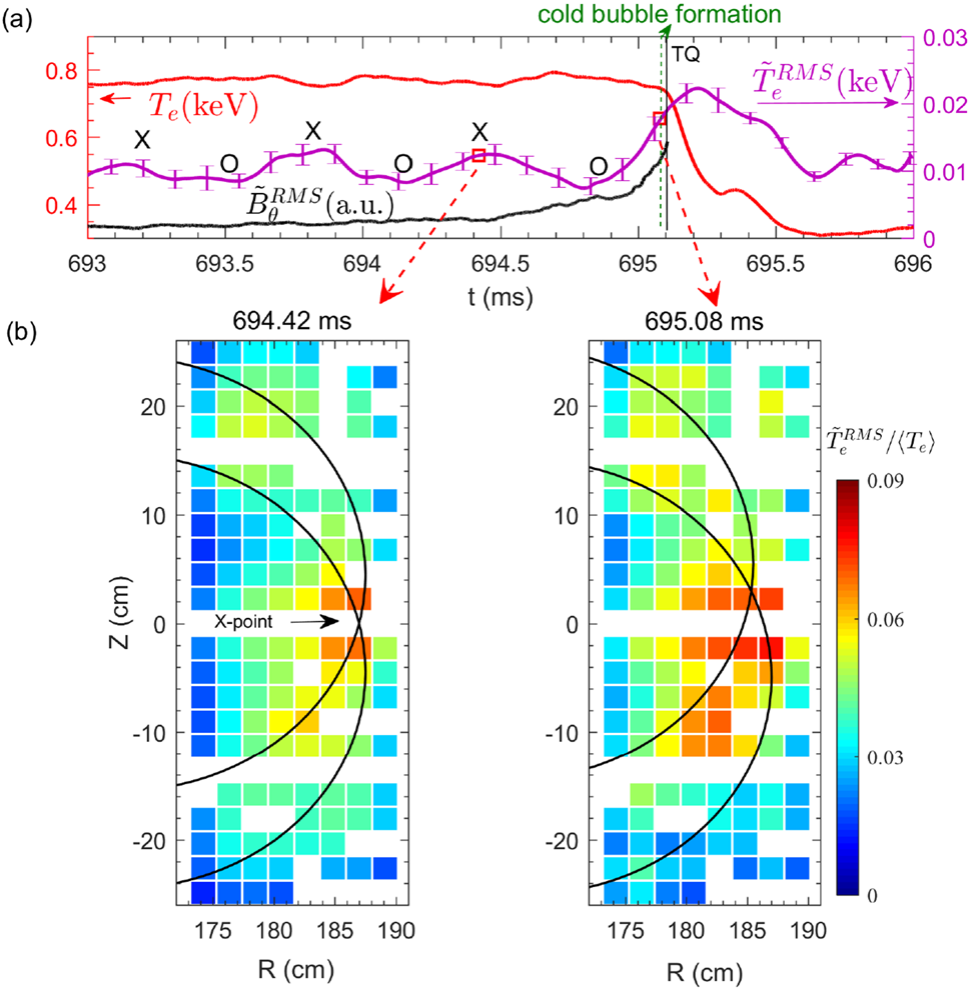
(**a**) Time evolutions of core $T_e$ (red curve), ${\tilde{T}}_e^{RMS}$ (purple curve) and ${\tilde{B}}_\theta ^{RMS}$ (black curve) signals. The ${\tilde{T}}_e^{RMS}$ is obtained by averaging serveral channels of $T_e$ fluctuations (15–100 kHz) measured around the island X-point. The ${\tilde{B}}_\theta ^{RMS}$ is the RMS value of $B_\theta$ fluctuations in the range of 15–400 kHz from the Mirnov coils mounted on the outbard midplane. (**b**) Two dimensional distribution of relative temperature fluctuations (${\tilde{T}}^{RMS}_e/\langle {T}_e\rangle$) in the frequency range of 15–100 kHz. The black curves denote the island separatrices (#33433).

In this experiment, similar drastic turbulence enhancement has also been observed when approaching the TQ of disruptive discharges without a small $T_e$ collapse preceding the TQ. The time evolutions of core $T_e$, ${\tilde{T}}_e^{RMS}$measured nearby the island X-point and edge ${\tilde{B}}_\theta ^{RMS}$ signals are plotted in Fig. 13a. Figure 13b illustrates the 2D distribution of the relative temperature fluctuations (${\tilde{T}}^{RMS}_e/\langle {T}_e\rangle$) at $t=694.42$ ms and $t=695.08$ ms prior to the TQ. In comparison with the first time ($t=694.42$ ms), the turbulence increases significantly at the second time ($t=695.08$ ms) nearby the X-point, and expands both radially and poloidally towards the lower 2/1 island O-point at *t*=695.08 ms. The increase of the electrostatic (${\tilde{T}}^{RMS}_e$) and magnetic fluctuation (${\tilde{B}}_\theta ^{RMS}$) amplitudes when approaching the TQ can also be seen in Fig. 13a. These results again signify that the increase of turbulence near the reconnection site plays an essential role in facilitating the TQ.

### Possible role of turbulence in facilitating the small collapse and the TQ

**Figure 14:**
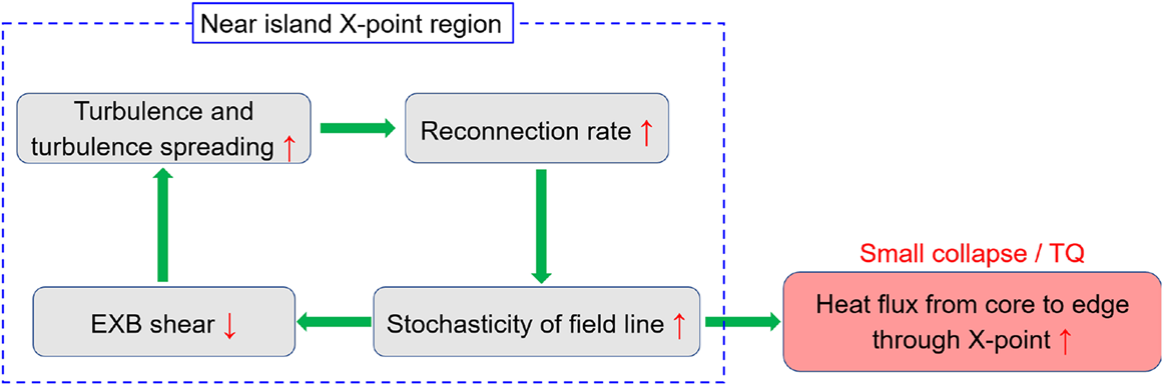
Diagrams of possible role of turbulence enhancement and related dynamics in facilitating the small collapse and the TQ.

In the “MHD instability dynamics towards the small collapse and the TQ” section, it has been shown that it is the formation of the *m* = 1 cold bubble along with its inward convection onto the central region that results in the TQ and subsequent plasma disruption. In the “Turbulence enhancement towards the small collapse and the TQ” section, substantial enhancement and expansion of turbulence nearby the island X-point are observed prior to the small central $T_e$ collapse and the TQ. The question is how does turbulence facilitate the small $T_e$ collapse and the TQ? The possible underlying physical mechanisms are presumed in Fig. 14.

At first, an enhancement of turbulence at the X-point region (e. g., due to the large pressure gradient) will increase the local resistivity dramatically, as reported in the magnetic reconnection experiment in laboratory plasmas^32^. This anomalous resistivity then increases the reconnection rate and makes the magnetic flux more stochastic. The enhancement of the field line stochasticity will bring two consequences. On one hand, it opens up additional outflow channels of the heat flux from the core to the edge through the island X-point, resulting in a reduction of $T_e$ before the small collapse or the TQ, as evidenced by the images in Figs. 7, 8. On the other hand, the enhanced magnetic stochasticity lessens the radial electric field $E_r$ and hence the $E_r\times B$ flow shear. The mechanism is similar to that testified in the resonant magnetic perturbation (RMP) experiments in TEXTOR^65^, MAST^66^, J-TEXT^67^ and DIII-D^68^, where the $E_r$ is generally reduced due to the parallel electron losses onto the plasma boundary and makes $E_r$ less negative or even positive^69^. The reduced $E_r\times B$ flow shear will lead to increase of turbulence and turbulence spreading, as predicted by theoretical models^62,63^. This process drives a feedback loop among the turbulence enhancement, reconnection rate increase, magnetic stochastization and $E_r\times B$ flow shear reduction.

As shown by ECE images in Figs. 7, 8, the cold bubble is only detected before the TQ, but not before the small collapse (see Fig. 7). On the other hand, turbulence spreading from the X-point to the O-point is much stronger prior to the TQ than before the small collapse, as seen in the ECE images in Figs. 10b, 13b. This suggests a possible link between turbulence spreading effects and cold bubble generation. Figures 7, 8 show that the cold bubble is developed in the inner region of the lower 2/1 island O-point (see image 9 in Fig. 7 and image 3 in Fig. 8). In Ref.^55^, the cold bubble is described as a current-carrying filament ($J_\parallel$). With turbulence spreading, the local turbulence magnitude inside the island increases, and consequently, a current filament can be generated in the stochastic zone ($k_\parallel \ne$ 0 with symmetry breaking) by $J_\parallel \propto \Sigma k_\parallel \langle {\tilde{n}}_e^2\rangle$^70,71^. In this view, the turbulence spreading into the island O-point is an essential element to generate the cold bubble, and subsequently, leads to the onset of TQ.

## Summary

In this work, the MHD instability dynamics and turbulence distribution have been studied in detail by the 2D ECEI diagnostic for the precursor phase of the HL-2A partial disruption. Two types of disruptive discharges have been found, one with a distinct stage of small central temperature collapse ($\sim$5–10%) around 1 millisecond before the TQ phase, while the other without. The results are summarized as follows:

1. For both types, a dominant 2/1 tearing mode is accompanied by a 1/1 internal mode which displaces the core plasma periodically during the island rotation in the precursor phase. For the small collapse and the TQ, the energy losses are both associated with an outflow of heat flux through the island X-point within 100–200 $\upmu$s before the core $T_e$ reduction.
2. The ECEI reveals that an *m*/*n* = 1/1 cold bubble is developed only before the TQ, but not before the small collapse. It is the generation of the cold bubble starting in the inner region of the lower 2/1 island O-point together with its inward convection that leads to the thermal quench and disruption.
3. The micro-scale electrostatic and electromagnetic fluctuations enhance towards the small collapse and more dramatically when approaching the TQ. In addition, strong turbulence spreading is observed prior to the TQ. The turbulence level and expansion cannot be fully explained by the linear stability analysis by GENE. Evidences suggest that nonlinear effects, such as reduction of $E_r\times B$ flow shear and turbulence spreading, may play an important role in the turbulence enhancement.
4. The observed turbulence at the reconnection region can result in the stochasticity of the magnetic field lines and decline of the flow shear, and thus, further increase of turbulence and turbulence spreading. Such a feedback loop facilitates the small collapse and the TQ. Moreover, the development of the cold bubble could be related to the explosive turbulence spreading onto the island O-point through $J_\parallel \propto \Sigma k_\parallel \langle {\tilde{n}}_e^2\rangle$, which is essential for the onset of the thermal quench.

These results deepen our understanding on the complicated process of interaction between MHD instabilities and turbulence before the plasma disruption, as well as provide general insight into the magnetic reconnection physics in magnetized plasmas.

## Data Availability

The datasets used and/or analysed during the current study available from the corresponding author on reasonable request.
